# Immunization with the Tc85-Derived Peptide Displayed on Virus-like Particles Confers Protection against *Trypanosoma cruzi* Infection

**DOI:** 10.1021/acsomega.6c04457

**Published:** 2026-08-11

**Authors:** Ugochukwu Oduwe, Giorgi Kenkebashvili, Syed Uddin, Edward Valencia Ayala, Gisele Macedo Rodrigues da Cunha, Jeffery Noble, Juan Jimenez Chunga, M. G. Finn, Alexandre Marques

**Affiliations:** † Center for Molecular and Cellular Biosciences, School of Biological, Environmental, and Earth Sciences, 679611 University of Southern Mississippi, Hattiesburg, Mississippi 39406, United States; ‡ Parasitología Molecular y Celular, Facultad de Ciencias Biológicas, 33209 Universidad Nacional Mayor de San Marcos, Lima 15081, Peru; § Departamento de Parasitologia, 28114 Universidade Federal de Minas Gerais, Belo Horizonte 31270-901, Brazil; ∥ School of Chemistry and Biochemistry, 1372 Georgia Institute of Technology, Atlanta, Georgia 30332, United States; ⊥ Parker H. Petit Institute of Bioengineering and Bioscience, 1372 Georgia Institute of Technology, Atlanta, Georgia 30332, United States; # School of Biological Sciences, 1372 Georgia Institute of Technology, Atlanta, Georgia 30332, United States

## Abstract

Chagas disease, caused by the *Trypanosoma cruzi*, remains a major global health burden with limited prophylactic options. We describe a vaccine platform that leverages a peptide epitope derived from the *T. cruzi* trans-sialidase group II (Tc85pep), displayed on the virus-like particle derived from the PP7 bacteriophage, to stimulate both innate and adaptive immunity. In silico analyses revealed that Tc85pep contains threonine residues predicted to undergo post-translational phosphorylation at conserved PKA and PKG sites and bind with high affinity to murine and human MHC class I and II alleles. Structural modeling and molecular docking suggested a potential interaction between Tc85pep and the TLR4/MD2 complex, supporting the hypothesis that the peptide may engage TLR4-associated signaling pathways. Functional analyses in TLR-deficient macrophages revealed that a multivalent conjugate of the peptide on the particle (PP7-Tc85pep) triggers nitric oxide production in a TLR4-dependent manner. In vivo, C57BL/6 mice immunized with PP7-Tc85pep exhibited a greater than 60% reduction in parasitemia and significant protection against cardiac inflammation and amastigote burden following a lethal *T. cruzi* challenge. The vaccine elicited a balanced Th1/Th2/Th17 immune response, marked by elevated detection of IL-10, IL-6, TNF-α, IFN-γ, and IL-17A, and provided tissue-level protection in the absence of chemotherapy. These findings identify PP7-Tc85pep as a potent, multifunctional epitope capable of engaging innate and adaptive immune pathways. The data support further development of Tc85pep-based epitope vaccines as a next-generation strategy for Chagas disease prevention.

## Graphical abstract

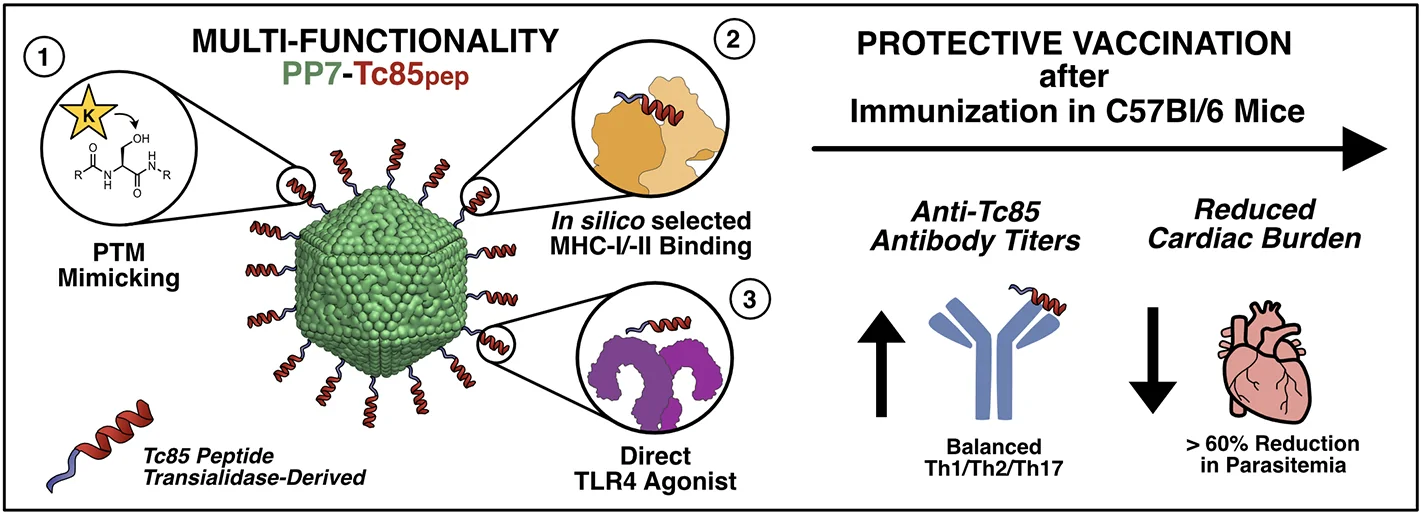

## Introduction

Chagas disease, caused by the protozoan parasite *Trypanosoma cruzi*, remains a significant public health concern in Latin America and is increasingly emerging in nonendemic regions such as the United States and Europe due to population mobility and climate change.^1,2^ With an estimated 6–8 million people infected globally and thousands of new cases annually, Chagas disease poses substantial social and economic burdens.^3,4^ While the acute phase often presents with mild or nonspecific symptoms, the chronic phase can lead to severe cardiac and gastrointestinal complications, including dilated cardiomyopathy and megacolon, often decades after the initial infection.^5,6^ Currently, the only approved treatments, benznidazole and nifurtimox, are limited by toxicity, variable efficacy in chronic stages, and the potential for treatment failure.^7,8^ These limitations underscore the urgent need for new therapeutic and preventive strategies, including vaccines.^9^

The surface of *T. cruzi* trypomastigotes is densely coated with glycosylphosphatidylinositol (GPI)-anchored glycoproteins, including mucins, mucin-associated surface proteins (MASPs), and members of the trans-sialidase (TS)/gp85 superfamily.^10,11^ Trans-sialidases, particularly Tc85, play a critical role in host-parasite interactions by facilitating parasite adhesion and invasion through interactions with host molecules such as cytokeratin 18, fibronectin, and laminin.^12–14^ Tc85 is a multifunctional glycoprotein with both adhesive and signaling properties, and it has been implicated in modulating host immune responses and enhancing parasite persistence through phosphorylation-driven activation of tyrosine kinase pathways.^12,15,16^ Importantly, Tc85 proteins contain multiple predicted B- and T-cell epitopes, making them attractive targets for immunological intervention and vaccine development.^17–19^

Vaccine strategies targeting *T. cruzi* have evolved from attenuated parasite preparations and recombinant proteins to epitope-based vaccines that leverage immunoinformatics for the selection of highly antigenic peptide regions.^18,20,21^ Peptide-based vaccines offer specificity and safety but often require effective delivery systems and immunostimulatory platforms to achieve protective immunity.^22,23^ Virus-like particles (VLPs), which mimic the structure of native viruses without containing infectious genetic material, represent a promising approach for enhancing the immunogenicity of synthetic peptides.^24–26^ VLPs self-assemble into nanoscale platforms that can present antigens in a repetitive, multivalent format, effectively stimulating B and T cell responses.^27,28^ Recent studies using VLPs displaying pathogen-derived peptides, including those from *T. cruzi*, have demonstrated their potential to induce strong humoral and cellular responses, as well as provide protection in murine models.^29,30^ In this study, we investigated the use of an immunogenic peptide derived from the Tc85 trans-sialidase protein, displayed on a self-adjuvanting VLP platform, to evaluate their immunogenic and protective potential in a murine model of Chagas disease. We assessed humoral immune responses as well as cytokine profiles, particularly the production of IL-6, IL-17, interferon-γ (IFN-γ) and tumor necrosis factor (TNF), which are key mediators of the Th1 immune response and crucial for parasite control.^31,32^ Our results contribute to the growing body of evidence supporting VLPs as an effective delivery system for peptide-based vaccines and identify Tc85-derived epitopes as promising targets for the development of next-generation Chagas disease vaccines.

## Materials and Methods

All experiments were approved and conducted according to the guidelines of the Ethics Committee on the Use of Animals (CEUA) of the Federal University of Minas Gerais (protocol n° 255/2013) and the Institutional Animal Care and Use Committee at the University of Southern Mississippi (Protocol 24021901).

### Mice

Female *Mus musculus* (C57BL/6 background), aged 6 to 8 weeks, genetically deficient in the α1,3-galactosyltransferase enzyme (αGalT-KO), were used in this study. Breeding and maintenance were conducted in the vivarium of the School of Biological, Environmental, and Earth Sciences, University of Southern Mississippi, and at the Department of Parasitology at the Federal University of Minas Gerais (UFMG). Animals were housed in pathogen-free standard 30.3 × 19.3 × 12.6 cm polypropylene cages (3–5 mice per cage) under controlled environmental conditions, including a 12-h light/dark cycle, regulated temperature, and humidity, and ad libitum access to species-specific commercial rodent chow and water. Female C57BL/6 wild-type, TLR2^–^/^–^, and TLR4^–^/^–^ knockout mice aged 6–8 weeks were obtained from The Jackson Laboratory and used in this study. Mice were euthanized by CO2 for all tissue recovery procedures following AVMA guidelines.

### Parasites

Trypomastigote forms of the *Trypanosoma cruzi* Y strain were maintained at the *T. cruzi* Biology Laboratory, Federal University of Minas Gerais (UFMG). The Y strain, classified as Discrete Typing Unit (DTU) TcII,^33^ was originally isolated in 1950 from a patient in the acute phase of Chagas disease in Marília, São Paulo, by Pereira de Freitas, and subsequently characterized by Silva and Nussenzweig.^34^ The Y strain was maintained through serial passage in mice, and bloodstream trypomastigotes were recovered for use in experimental challenges.

### Peptide Design and Bioinformatic Analysis

The immunogenic peptide Tc85pep was derived from *Trypanosoma cruzi* trans-sialidase surface glycoprotein Tc85–11 (TSGII; GenBank: AF085686.1). The selection was informed by predictive analyses of post-translational modifications, particularly threonine phosphorylation. NetPhos 3.1 (https://services.healthtech.dtu.dk/services/NetPhos-3.1/) was employed to identify consensus phosphorylation sites, yielding high prediction scores for protein kinase A (PKA, 0.79) and protein kinase G (PKG, 0.58). To evaluate Tc85pep’s potential to stimulate adaptive immune responses, epitope prediction was performed using multiple computational platforms. Linear B-cell epitopes were identified using ABCpred,^35^ while class I and class II T-cell epitope mapping was performed with NetMHCpan and NetMHCIIpan, respectively.^36,37^ The peptide was predicted to bind with high affinity to murine MHC class I (H-2-Dd) and a range of human HLA alleles, including HLA-A01:01, HLA-B35:01, and HLA-C*07:02, suggesting broad immunogenic potential. Structural modeling of Tc85pep was performed with PEP-FOLD 2.0 and validated with SWISS-MODEL. Molecular docking was performed using the murine TLR4/MD-2 complex (PDB ID: 5ijc),^38^ corresponding to the agonist-bound conformation. The cocrystallized ligand (neoseptin-3) was removed prior to docking, and the structure was prepared by removing heteroatoms, adding hydrogens, and optimizing protonation states. using ClusPro. The docking simulations indicated favorable interactions with the myeloid differentiation factor 2 (MD-2) hydrophobic cavity, consistent with predicted TLR4 agonistic activity.^38^ The agonist-bound structure was selected to better represent the active conformation of TLR4/MD-2 relevant for ligand-induced signaling.

### Virus-like Particle Peptide Display

#### Peptide and Detection Reagent Synthesis

The -GS-(Tc85–11_594_) peptide (Pra-GS-TTRRSTWELKKEYQV,^39^ designated **Tc85pep**) was synthesized by standard Fmoc solid-phase peptide synthesis on Rink Amide resin (loading: ∼ 0.4–0.8 mmol/g) using an automated peptide synthesizer (CS Bio II, Menlo Park, CA). Synthesis was performed at a 0.1–0.2 mmol scale in N,N-dimethylformamide (DMF) using N,N′-diisopropylcarbodiimide (DIC) and ethyl 2-cyano-2-(hydroxyimino)­acetate (Oxyma) as coupling agents. Serial Fmoc deprotection was carried out using 20% piperidine in DMF with standard coupling and deprotection cycles programmed on the synthesizer.

Following synthesis, the resin was washed extensively with CH_2_Cl_2_ and MeOH, then dried under vacuum. Global deprotection and resin-cleavage was performed using 10 mL of a cleavage cocktail consisting of 95% trifluoroacetic acid, 2.5% triisopropylsilane, and 2.5% water (v/v) for 2 h with shaking at room temperature. The cleavage mixture was filtered and the peptide precipitated by the addition of cold (−78 °C) diethyl ether and pelleted by centrifugation at 4200 rcf for 10 min. The pellet was washed three more times with additional cold ether to remove residual scavengers and TFA. Pellets were dried under vacuum and stored at −20 °C until use. No further purification of the peptide was necessary for VLP modification.

The biotinylated detection reagent **2** (Figure 2) was generated by conjugation to biotin-PEG_3_-azide (Lumiprobe, Westminster, MD, USA) on an ∼4 mg peptide scale. The peptide was clicked in slight excess (1.33 equiv) relative to biotin-azide in the presence of copper sulfate, tris­(3-hydroxypropyl-4-triazolyl)­methyl)­amine (THPTA), aminoguanidine, and sodium ascorbate, as previously described.^40^ Successful conjugation was confirmed in the neat reaction mixture via ESI-ToF mass spectrometry. Following the reaction, EDTA (10-fold molar excess relative to copper) was added directly to chelate residual copper, and any precipitated solids were pelleted by centrifugation at 21,000 × g. The supernatant was sterile-filtered through a 0.22 μm membrane filter and stored at −20 °C until use. Free biotin was not detected at appreciable levels, and therefore the neat mixture was used directly as the detection reagent, omitting HPLC purification to preserve yield.

#### Expression and Purification of PP7 Virus-like Particles

Virus-like particles derived from the PP7 bacteriophage were produced and characterized as previously described;^41^ note that the particle made from monomeric subunits was employed, which is dominated by T = 3 icosahedral particles approximately 27 nm in diameter.^42^ Briefly, chemically competent BL21 (DE3) *E. coli* cells were transformed with the coat-protein (CP)-containing pET28a plasmid according to manufacturer’s instructions. Transformed cells were plated onto selective 2YT-agar plates and grown overnight at 37 °C. The day before expression, a single colony was inoculated into super optimal broth (SOB) for overnight growth at 37 °C. The following morning, expression cultures were inoculated to a larger flask and grown to an optical density (600 nm) of 0.6–0.9 before induction with 1 mM IPTG. Cells were maintained at 30 °C for 16 h and then harvested by centrifugation (JA-10 rotor, 6k rpm, 10 min).

Cell pellets were resuspended in 1x PBS and lysed by sonication (8 min total, 50% power, 5 s intervals, 4 °C ice bath). Lysate was clarified by centrifugation (JA-17 rotor, 14k rpm, 10 min, 4 °C). Protein was then salted out of the clarified lysate with 27% ammonium sulfate (1–2 h, 4 °C) before another round of centrifugation. The resulting pellet (derived from 500 mL expression culture) was solubilized with 8 mL of PBS buffer (137 mM NaCl, 2.7 mM KCl, 10 mM Na_2_HPO_4_, 1.8 mM KH_2_PO_4_, pH 7.4), followed by the addition of 8 mL of a 1:1 n-butanol:chloroform mixture to remove lipids and other nonpolar material. This mixture was agitated to mix, then centrifuged to separate the immiscible layers (JA-17, 14k rpm, 10 min, 4 °C). The top, VLP-rich, aqueous layer was collected and loaded onto a 10–40% sucrose density gradient for further purification. Ultracentrifugation (SW32 rotor, 28k rpm, 4 h, 4 °C) provided a VLP-rich band, which was extracted and pelleted by ultracentrifugation (70.1 Ti rotor, 68k rpm, 2 h, 4 °C). The sequential organic extraction and sucrose gradient purification steps remove the vast majority of potential endotoxin contaminants, as previously verified by endotoxin assay. The resulting pellet was resuspended in PBS, sterile filtered through a 0.22 μm syringe filter, and stored at 4 °C for characterization and subsequent use.

#### Peptide Bioconjugation of Virus-like Particles

Reactive VLP surface amines were first modified with an azide-containing *N*-hydroxysuccinimide ester linker (**1**, Figure 1, 10 equiv. relative to coat protein, 1.25 equiv. relative to primary amine groups; VLP concentration 8 mg/mL) in 10% DMSO. The reaction was allowed to proceed with gentle shaking at room temperature for 4 h, providing an average of approximately 330 attached azide groups per particle as determined by ESI-ToF mass spectrometry. The particles were separated from small molecules with a 2 mL Zeba 7k MWCO cutoff desalting column. The Tc85Pep peptide was then attached under standard CuAAC conditions using 5 equiv of peptide-alkyne and VLP concentration of 5 mg/mL. The CuAAC reaction was quenched by the addition of EDTA (10-fold molar excess relative to Cu), the solution was filtered through a 0.22 μm filter, and the VLP conjugate was purified by size-exclusion FPLC (Superose 6, Increase 10/300 GL column, 0.4 mL/min PBS, 2 mL fraction collection). The VLP-containing fractions were collected and concentrated with a 100 kDa MWCO filter (Amicon) and 0.22 μm sterile-filtered again before final characterization. Protein recovery was determined via Bradford assay, and particles were characterized by dynamic light scattering for hydrodynamic radii and microfluidic denaturing gel electrophoresis (Agilent Bioanalyzer, Protein 80 chip) for composition.

**Figure 1:**
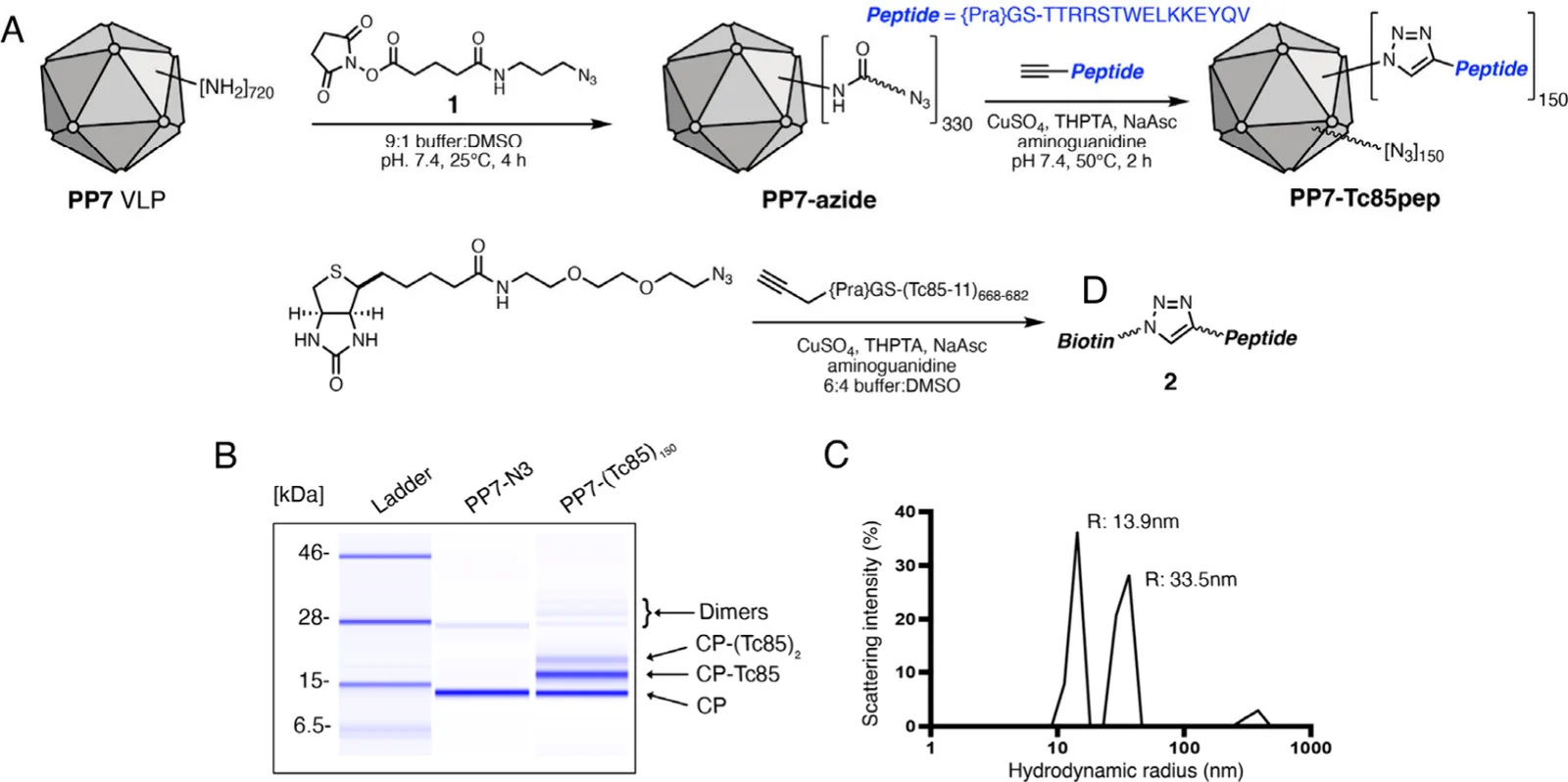
Tc85_593_-Displaying PP7VLPs. A) Surface Labeling of PP7 with the Tc85 Antigenic Peptide. B) Electrophoretic analysis of the final peptide conjugate for quantitation of particle labeling. C) Dynamic light scattering. D) Schematic representation of the biotin conjugation strategy used for peptide functionalization prior to VLP coupling.

### ELISA

Blood samples were collected via submandibular puncture before the first immunization and 7 days after the final immunization dose (first or second, depending on the protocol). At the time of euthanasia, bulk blood collection was performed via cardiac puncture. All blood samples were collected into tubes without anticoagulant, and sera were separated by centrifugation at 3,000 × g for 15 min at 25 °C, then stored at −20 °C until analysis. For the immunoenzymatic assay, 96-well polystyrene microplates (NUNC MaxiSorp, Thermo Fisher Scientific) were coated with 50 μL of PP7-Tc85pep, Biotin-Tc85pep (used with streptavidin-coated plates), or PP7-WT particles at a concentration of 5 μg/mL in 100 mM carbonate-bicarbonate buffer (pH 9.6) and incubated for 18 h at 2–8 °C. Plates were then blocked with 100 μL of PBS containing 1% bovine serum albumin (PBS1X–BSA 1%) for 50 min at 37 °C. Following blocking, 50 μL of diluted serum (1:100–1:1600) in PBS1X–BSA 1% was added to each well in triplicate and incubated for 90 min at 37 °C. Plates were washed three times with 200 μL of PBS containing 0.05% Tween-20 (PBS1X–T 0.05%) per well (Agilent BioTek 50 TS). Next, 50 μL of biotin-conjugated antimouse IgG (Invitrogen), diluted 1:4,000 in PBS1X–BSA 1%, was added and incubated for 50 min at 37 °C. After three additional washes, 50 μL of streptavidin-horseradish peroxidase (GE Healthcare), diluted 1:3,000 in PBS1X–BSA 1%, was added and incubated for 50 min at 37 °C. Following a final washing step, 50 μL of substrate solution (Ultra-TMB-ELISA, Thermo) was added to each well and incubated for 30 min in the dark. 50 μL of the stop solution (4N sulfuric acid) was added, and absorbance was measured at 490 nm using a microplate reader (Agilent BioTek 800 TS).

### Peritoneal Macrophage Activation Assays in TLR-Deficient Mice

Peritoneal macrophages were isolated from C57BL/6 wild-type, TLR2^–^/^–^, and TLR4^–^/^–^ knockout mice. Mice received intraperitoneal injections of 2 mL of 3% sterile thioglycolate. After 72 h, peritoneal exudate cells were harvested by lavage with ice-cold RPMI 1640 medium. Cells were seeded at 3 × 10^5^ cells/well in 96-well plates and allowed to adhere for 1 h at 37 °C, 5% CO_2_. Cultures were maintained in RPMI 1640 supplemented with 2 mM l-glutamine, 50 U/mL penicillin, 50 μg/mL streptomycin, and 10% fetal bovine serum (FBS). Macrophages were primed with recombinant murine IFN-γ (3 IU/mL) for 18 h and then stimulated with lipopolysaccharide (LPS), PP7-Control, or PP7-Tc85pep and media alone (10 μg/mL). Nitric oxide (NO) production was quantified by measuring nitrite concentrations using the Griess Reagent System (Promega).

### Cytokines

Immediately following euthanasia, the spleen was collected from each mouse and homogenized in 1 mL of PBS buffer supplemented with protease inhibitors. Tissue homogenates were centrifuged at 10,000 rpm for 10 min at 4 °C, and the resulting supernatants were collected and stored at −80 °C until analysis. Cytokine levels of IL-6, IL-17 (BioLegend ELISA Max), TNF-a, and IFN-γ (Invitrogen) were quantified following the manufacturer’s instructions. Assays were performed using a standard capture ELISA format.

### Immunization and Challenge

Female aGalT-KO C57BL/6 mice (6–8 weeks old) were housed under specific pathogen-free (SPF) conditions at the School of Biological, Environmental, and Earth Sciences, University of Southern Mississippi animal facility, with free access to food and water. While α-Gal is not used here as an antigen, the anti-α-Gal immune response is relevant to the mouse model of Chagas disease.^43^ These mice (which, like humans, do not present α-Gal as a self-epitope) were therefore used as the most translationally relevant rodent model available. Mice (n = 6 per group) were immunized subcutaneously at a single injection site in the right flank with 20 μg of PP7-Tc85pep virus-like particles (VLPs) in 100 μL sterile PBS. A booster dose was administered 14 days later using the same formulation. Control groups received PP7-Control (nonfunctionalized VLPs) or PBS. One week after the boost, all mice were challenged intraperitoneally with 1 × 10^4^ bloodstream trypomastigotes of the *T. cruzi* Y strain (DTU TcII), previously isolated from donor mice. Following infection, parasitemia, immune responses, and tissue pathology were monitored and analyzed.

### Evaluation of Infection and Pathology

Parasitemia was evaluated by microscopic analysis of 5 μL of peripheral blood samples collected via tail-vein puncture. Trypomastigotes were counted in 50 random microscopic fields at 40× magnification and reported as parasites/mL. On day 40 postinfection, six mice per group were euthanized. Hearts were harvested, fixed in 10% neutral-buffered formalin, embedded in paraffin, and sectioned at 4 μm. Hematoxylin and eosin (H&E) staining was performed. Histological scoring included assessment of: (i) inflammation (Grade 0–3), (ii) degenerative changes (necrosis/autolysis, grade 0–3), (iii) cardiomyocyte hypertrophy (Grade 0–3), and (iv) parasitic load quantified by the number of amastigote nests per 10 fields at 20× magnification. Image analysis was conducted using Motic Images Plus 2.0 software.

### Statistical Analysis

Statistical analysis was performed using GraphPad Prism v7.0. Parasitemia and weight change were analyzed via two-way ANOVA followed by Bonferroni’s post hoc test. Kaplan–Meier survival curves were compared using the Log-Rank (Mantel–Cox) test. For comparisons between two groups, Student’s *t* test was used when data met normality assumptions; otherwise, the Mann–Whitney U test was applied. For multigroup comparisons, one-way ANOVA with Tukey’s post hoc test was used for parametric data, and the Kruskal–Wallis test with Dunn’s post hoc test was applied for nonparametric data. Data normality was assessed using the D’Agostino–Pearson test. Differences were considered statistically significant at *p* < 0.05.

## Results

### *In Silico* Prediction and Peptide Characterization TLR-Dependent Activation by Tc85pep

A linear B-cell epitope (TTRRSTWELKKEYQV), designated Tc85pep, was identified within the surface glycoprotein Tc85–11 of *Trypanosoma cruzi* using immunoinformatic analysis. Tc85pep contains a threonine-rich motif with high phosphorylation scores (NetPhos PKA score 0.79; PKG score 0.58), indicating a potential role for post-translational modification in immunogenicity (Figure S1 and Figure S2). Such phosphorylation may have functional significance, as Chuenkova and colleagues demonstrated that the *T. cruzi* trans-sialidase undergoes Akt-mediated serine/threonine phosphorylation, both intracellularly and via surface receptor engagement, thereby promoting antiapoptotic signals in Schwann cells and facilitating parasite survival.^44^ Epitope prediction analyses suggested potential binding across murine and human MHC alleles, including overlapping CD4^+^ and CD8^+^ T-cell epitope cores. Antigenicity analysis using VaxiJen v2.0 yielded a score of 0.66, supporting the predicted immunogenic potential of the peptide (Table S1, Table S2, and Table S3). Structural modeling of the Tc85–11 protein with I-TASSER^45,46^ yielded a moderately reliable result (C-score = −0.49, TM-score = 0.65 ± 0.13, RMSD = 9.5 ± 4.6 Å) that closely matched the predicted structure in the AlphaFold database^47,48^ (AF–O77209-F1-v4, in which the Tc85pep epitope was found in a region of high predictive confidence). Tc85pep is expected to be surface-exposed in an accessible conformation. These findings suggested that the Tc85-derived peptide would be a promising target for vaccine development and serological applications.

For TLR4 binding, we performed in silico structural modeling of Tc85pep, *which* revealed a β-turn conformation, and docking analyses showed a stable interaction within the MD2 pocket of the murine TLR4/MD2 complex, supplementing the hypothesis that Tc85pep may function as a TLR4 agonist (Figure 2A,**B**). Although these findings do not demonstrate direct binding or stable occupancy of the MD2 cavity, they support the possibility that TC85 may engage TLR-associated signaling pathways. Consistent with this hypothesis, macrophages derived from TLR2^–^/^–^ knockout mice produced significant levels of NO in response to PP7-Tc85pep stimulation compared to negative controls (media and PP7-WT), whereas macrophages from TLR4^–^/^–^ mice did not (<5 μM NO, Figure 2C), confirming the dependence of peptide-mediated activation on TLR4 signaling.

**Figure 2:**
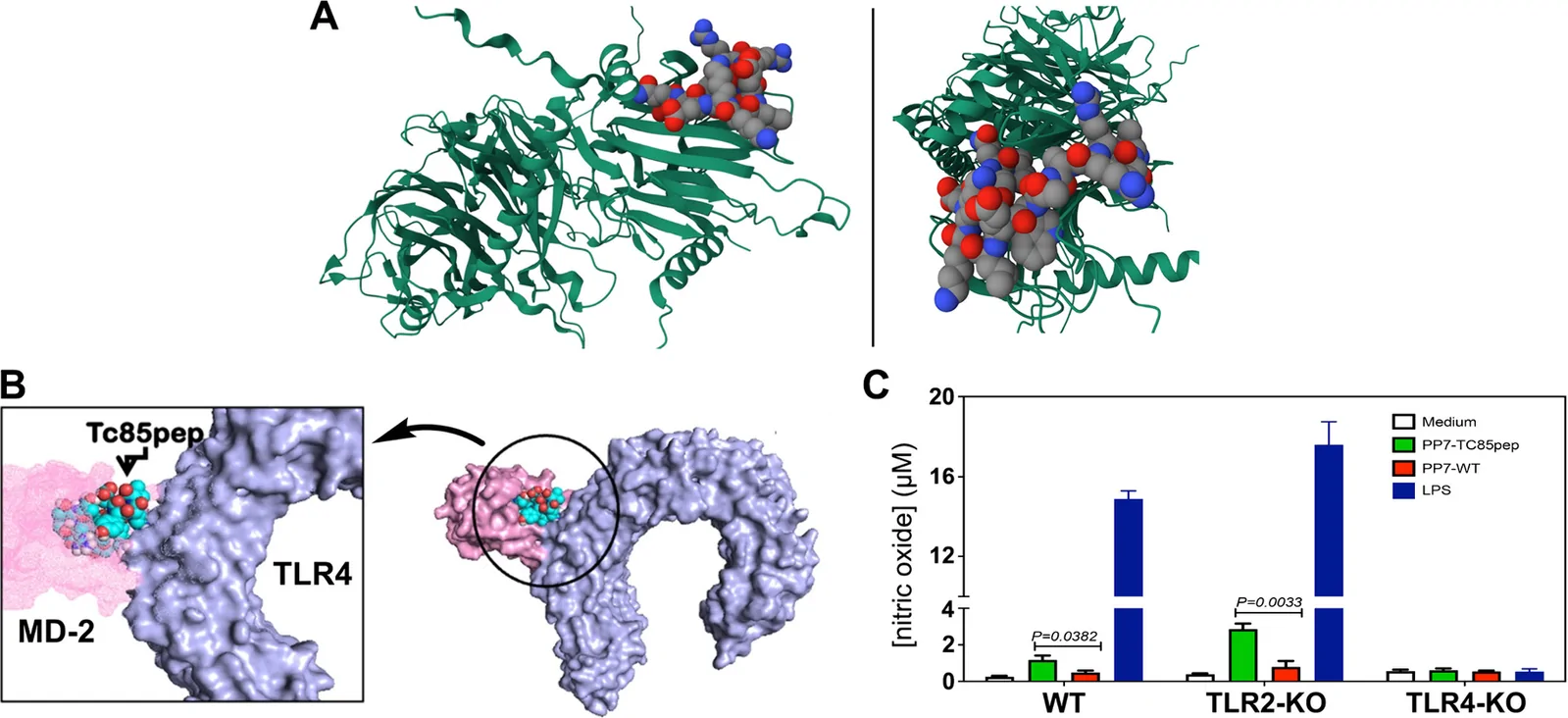
(A) Predicted structure of the Tc85–11 protein (GenBank AAD13347–1, UniProt 077209), with the target peptide epitope in space-filling representation; (*left*) the central core region of the protein; (*right*) view rotated to look down onto the peptide. (B) CLUSPRO modeling of the interaction between Tc85pep and the MD2-TLR4 complex. The TLR4/MD-2 structure shown corresponds to the agonist-bound conformation (PDB 5ijc) after removal of the cocrystallized ligand. This interaction signals macrophage activation and the production of pro-inflammatory cytokines. (C) Nitric oxide production by stimulated peritoneal macrophages isolated from wild-type (WT), TLR2^–/–^, and TLR4^–/–^ mice. Macrophages were stimulated *in vitro* with the indicated agents at a concentration of 1 μg/mL. “Medium” refers to unstimulated control cells cultured in medium alone. Error bars = standard deviation from 3 independent experiments.

### Vaccinated Mice with Tc85pep Displayed on Virus-like Particles Produce High Titers of IgG Antibodies

Tc85pep was synthesized with the addition of an alkyne-containing amino acid at the N-terminus, allowing for convenient and efficient conjugation to PP7 virus-like particles functionalized at surface lysine residues with azide groups using a short amine-reactive connector. An average of 148 peptides was attached per particle; the resulting VLPs (designated PP7-Tc85) were characterized by standard methods and showed no tendency to aggregate in solution (Figure 1). Mice were immunized with PP7-Tc85 and boosted with the same dose 2 weeks later, eliciting a clear antipeptide IgG response (Figure 3; serum titer = 3.2 × 10^3^ at week 3, Supporting Information Figure S3), indicating that the conjugated peptide is immunogenic and effectively presented by the VLP platform.

**Figure 3:**
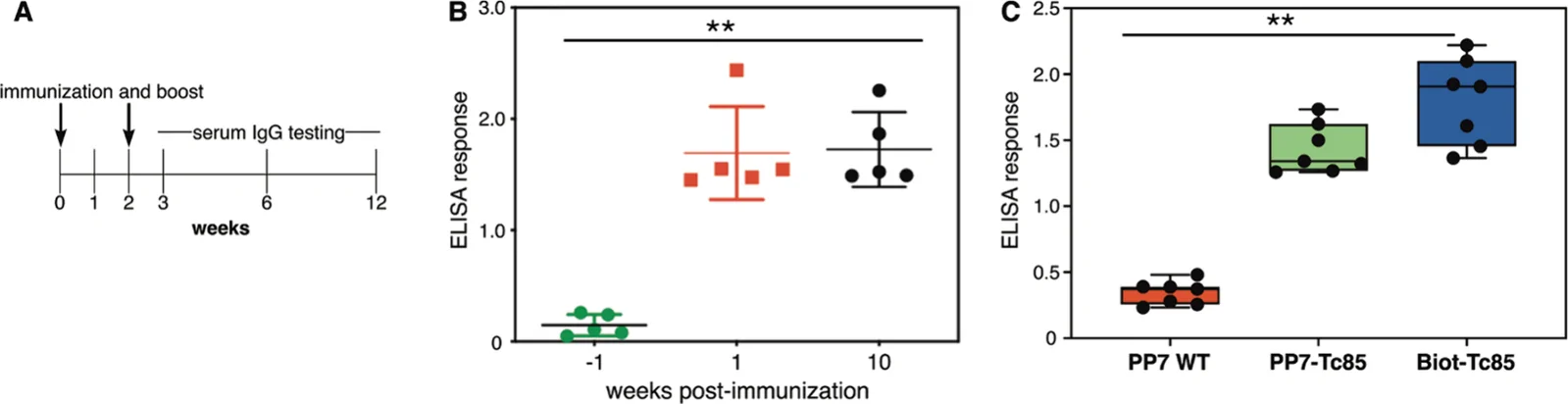
**Humoral immune response following immunization and antigen specificity analysis**. (A) Experimental timeline; dose = 20 μg given at weeks 0 and 2. Serum samples were collected and analyzed up to 10 weeks postimmunization (B) Abs (492 nm) in serum IgG ELISA assay against plated biotinylated Tc85pep (Biot-Tc85) for serum taken at the indicated time points. (C) Specificity analysis of immune sera by ELISA (week 3, serum dilution 1:400), assayed against plated PP7-WT (negative control), the PP7-Tc85 immunogen, and biotinylated Tc85 peptide. Data represent individual animals (*n* = 6–8 per group). ** *P* < 0.001.

### Cytokines IL-10, IFN-γ, TNF-α, IL-6, and IL-17 Production

Spleens collected from immunized mice 2 weeks after boost were analyzed for cytokine levels in homogenized tissue in order to capture the cytokine microenvironment at a defined memory/effector time point. This approach assesses the integrated output of multiple immune cell populations (T cells, NK cells, macrophages, dendritic cells) within the spleen architecture. By sampling 14 days postboost, we aimed to coincide with the plateau of secondary immune activation and contraction, presumably the cytokine milieu that most influences B and T cell differentiation.^49,50^ Significant elevation of pro-inflammatory (IFN-γ, TNF-α, IL-6, IL-17) and regulatory (IL-10) cytokines was observed in animals vaccinated with PP7-Tc85pep compared to those immunized with control VLPs (Figure 4). This type of balanced response has been found to help limit parasite replication while preventing excessive tissue damage and inflammation, thus potentially enhancing protection against Chagas disease progression.^51^

**Figure 4:**
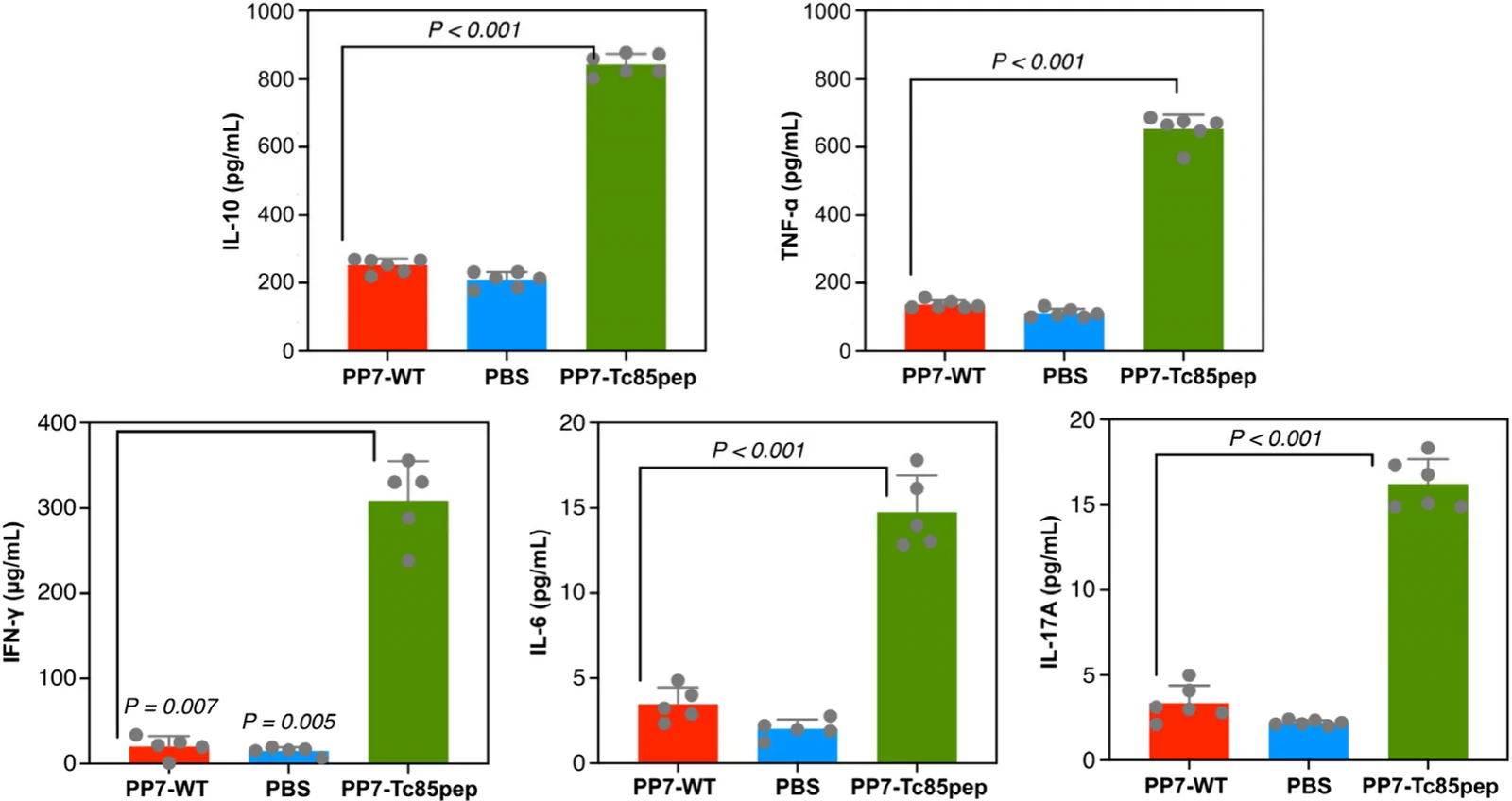
Cytokine response following PP7-Tc85pep immunization. Mice were immunized subcutaneously with 20 μg of PP7-Tc85pep or unfunctionalized (WT) VLPs, followed by a booster dose 14 days later. Spleens were harvested 14 days after boost for cytokine analysis compared to the control (buffer only) group. Data represent mean ± standard error from 5 to 7 animals per group.

### Immunization Efficacy and Parasitemia Control

Mice immunized and boosted with PP7-Tc85pep exhibited significantly lower levels of circulating trypomastigotes compared to mice receiving PP7 alone or PBS (Figure 5A). On day 7 postinfection, the average peak parasitemia (trypomastigotes/mL) in the PP7-Tc85 group reached 37% of the level observed in the PP7-WT control group and 28% of the parasitemia count in the unvaccinated PBS group, although the large variance in the latter two groups made the values statistically significant only to the 90% confidence level. By day 9, parasitemia was nearly undetectable in the PP7-Tc85 group, indicating strong protection during the acute phase of infection (Figure 5A). Furthermore, vaccinated mice maintained body weight more consistently, while the infected, unvaccinated control displayed progressive weight loss over time (Figure 5B). Histopathological analysis corroborated these findings. Hematoxylin and eosin (H&E)-stained cardiac sections from uninfected controls showed normal myocardial architecture without inflammation (Figure 5C). Infected, unvaccinated mice displayed severe myocardial damage with parasite nests and extensive inflammatory infiltrates (Figure 5D). In contrast, hearts from PP7-Tc85-vaccinated mice exhibited well-preserved tissue with minimal inflammation and only rare parasite foci (Figure 5E), demonstrating that vaccination effectively mitigated *T. cruzi*-induced cardiac pathology.

**Figure 5:**
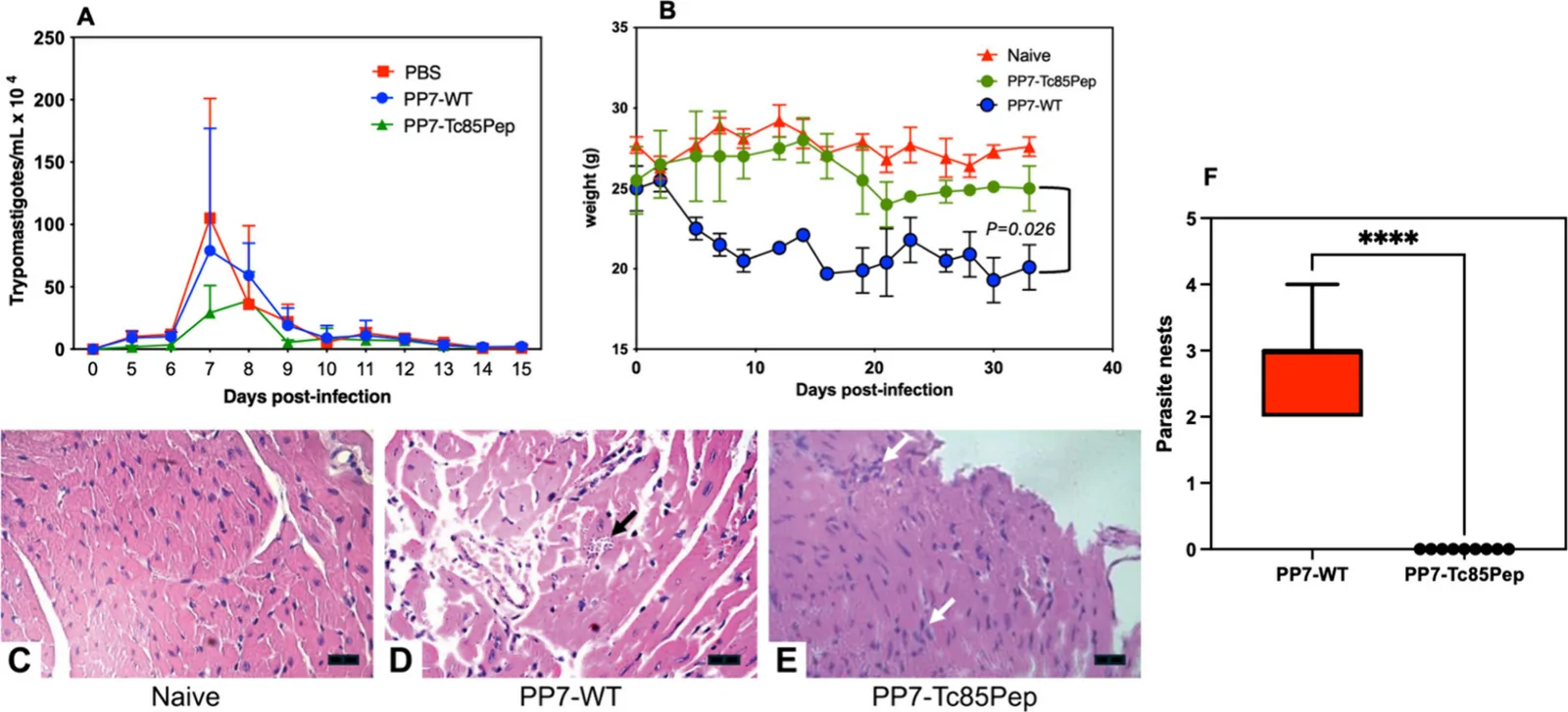
**PP7-Tc85pep protection against pathogen challenge**. (A) Parasitemia from experimental mice infected with *T. cruzi* Y strain. (B) Mouse weights. Longitudinal mixed-effects analysis demonstrated significant effects of time and treatment group on body weight kinetics, with a significant group × time interaction (*p* = 0.026), indicating that PP7-Tc85pep-immunized mice maintained a more stable weight profile throughout infection compared to control animals. (C-E) Histological analyses (hematoxylin and eosin staining) of representative sections of heart tissue: C = naïve (noninfected) mice; D = vaccinated and boosted with PP7-WT and infected at 1 week postbooster; E = vaccinated and boosted 14 days after the first dose with PP7-Tc85pep and infected at 1 week postbooster. The arrow in panel D marks an amastigote nest; only tissues corresponding to panel D showed such amastigote nests, which were completely absent in all tissues corresponding to panel E. Arrows in panel E mark potentially inflammatory infiltrates in the myocardium. Scale bar = 16 μm. (F) Quantification of amastigote nests in cardiac tissue. Around 40 microscopic fields per heart section were examined per animal (*n* = 8 per group) on H&E-stained tissue collected 21 days postinfection with *T. cruzi* (Y strain), from mice vaccinated and boosted with PP7-WT or PP7-Tc85pep. Amastigote nests were absent in all PP7-Tc85pep-immunized animals. Data are presented as mean ± SD; ****p < 0.0001 by unpaired *t* test.

## Discussion

In this study, we present evidence that a single predicted phosphorylatable peptide, when displayed on virus-like particles (VLPs), induces innate and adaptive immune responses that significantly attenuate acute *Trypanosoma cruzi* infection and ameliorate cardiac pathology in C57BL/6 mice. The immune response to the peptide drives reductions in parasitemia of over 60% on day 9 postinfection without adjunct chemotherapy and markedly diminishes myocardial inflammation and parasite load by day 21. These results position Tc85pep among a growing group of promising monovalent subunit vaccine candidates for Chagas disease, demonstrating protective effects comparable to those reported in more complex multicomponent vaccination strategies and therapeutic approaches.^52–54^

The *in vitro* macrophage assays reported here emphasize the mechanistic specificity of this response: in wild-type and TLR2^–^/^–^ macrophages, PP7-Tc85pep triggered the production of substantial amounts of nitric oxide, whereas TLR4^–^/^–^ macrophages were unresponsive, confirming that Tc85pep-mediated macrophage activation is TLR4 dependent. Molecular docking analysis suggested a potential interaction between Tc85pep and the TLR4/MD2 complex, although the physiological relevance and stability of this interaction remain to be experimentally validated. TLR4 is a pattern recognition receptor on antigen-presenting cells that recognizes structurally diverse ligands and can induce cytokine production, including TNF-α and IL-6.^55^ We suggest that TLR4 engagement is critical, as it primes the innate immune system, and the induction of macrophage-derived NO can enhance early parasite control even before adaptive immunity fully develops.^56–58^

Tc85pep contains a threonine-rich region that, according to NetPhos 3.1, is highly amenable to phosphorylation at consensus PKA and PKG motifs (scores: 0.79 and 0.58, respectively). Phosphorylation-dependent regulation of trans-sialidase proteins has been implicated in host–parasite interactions and parasite persistence, although distinct kinase families such as Akt/PKB, PKC, PKA, and PKG recognize different substrate consensus motifs and may regulate different functional contexts. Thus, the predicted phosphorylation propensity of Tc85pep should be interpreted cautiously as a potential indicator of post-translational regulatory capacity rather than evidence of a specific kinase-mediated mechanism.^59,60^ Recent studies suggest that epitope-based vaccines incorporating phosphorylated peptides^61,62^ and adjuvants^63–66^ may engage innate immune receptors to enhance adaptive immunity. We have no evidence to suggest that the VLP-displayed peptide gets phosphorylated after administration, but the possibility that such post-translational modifications contribute to immune modulation is interesting. It is also possible that antibodies recognizing the nonphosphorylated peptide may be able to block phosphorylation of Tc85 *in vivo*, and thereby hinder parasite persistence as proposed by Chuenkova.^44^

Our adaptive immune data showed expanded IL-10, IL-6, TNF-α, IFN-γ, IL-17A, and antibody responses consistent with a Th1/Th2/Th17 balanced profile, an optimal combination for controlling the pathology of intracellular protozoa such as *T. cruzi*.^67,68^ This cytokine milieu supports macrophage activation and granulopoiesis while reducing the risk of excessive inflammation and tissue damage commonly linked to pure Th1-dominated responses. Such balanced immunity is reminiscent of what has been observed with parasite-derived neurotrophic factor (PDNF) mediated host cell protection, although in our case, the outcome is parasite clearance rather than cell survival.

The elicitation of both CD4^+^ and CD8^+^ T cell responses, as predicted via *in silico* MHC binding analysis, further supports the peptide’s capacity to promote broad cellular immunity. Compared to previous approaches involving trans-sialidase- or Tc24-based vaccines, which may require combination with benznidazole to achieve protection,^69^ Tc85pep demonstrated strong efficacy in a simplified peptide–VLP platform without chemotherapeutic support.^70–72^ This streamlined construct is considerably easier to prepare and characterize and is highly stable, making progression to larger animal models and eventual clinical trials more feasible.

In summary, we have combined immunoinformatics, structural modeling, docking studies, and immunological and *in vivo* assessments to introduce PP7-Tc85pep as a simple, novel VLP-based peptide vaccine. It proved capable of activating TLR4-dependent innate immunity, eliciting balanced Th1/Th2/Th17 immune responses, and conferring substantial protection against acute Chagas disease. While additional studies will be required to fully elucidate the underlying mechanisms, the current findings highlight the utility of rational peptide design combined with VLP-based delivery to generate protective immunity against *T. cruzi*. This work also raises the possibility that peptides harboring predicted phosphorylation motifs may constitute a potent class of immunomodulatory vaccine candidates, warranting further exploration in other parasitic diseases.

## Supplementary Material



## Notes

Prediction of threonine phosphorylation sites in the Tc85–11surface glycoprotein of *Trypanosoma cruzi* using NetPhos 3.1, Conservation and immunogenic potential of the RRSTW phosphorylation motif across *Trypanosoma cruzi* trans-sialidase family members, and Bioinformatics prediction and peptide localization (PDF)
