# Supplementary material for: Immunization with the Tc85-Derived Peptide Displayed on Virus-like Particles Confers Protection against Trypanosoma cruzi Infection
Source: ACS Omega. 2026 Aug 11;11(33):49775–84. doi: 10.1021/acsomega.6c04457 (PMC13519998; doi:10.1021/acsomega.6c04457)

Supporting Information

Immunization with Tc85-Derived Peptide Displayed on Virus-Like Particles Confers Protection Against *Trypanosoma cruzi* Infection

Ugochukwu Oduwe[1], Giorgi Kenkebashvili[1], Syed Uddin[1], Edward Ayala[2], Gisele Macedo Rodrigues da Cunha[3], Jeffery Noble[4,5], Juan Chunga[2], M.G. Finn[4,6] and Alexandre Marques[1*]

[1] Center for Molecular and Cellular Biosciences, School of Biological, Environmental, and Earth Sciences, University of Southern Mississippi, Hattiesburg, MS, USA;
[2] Universidad Nacional Mayor de San Marcos, Faculdad de Ciencias Biologicas, Escuela Profesional de Microbiologı´a y Parasitologı´a, Laboratorio de Parasitologıa em Fauna Silvestre y Zoonosis, Lima, Peru;
[3] Universidade Federal de Minas Gerais, Departamento de Parasitologia, Belo Horizonte, Brazil.
[4] School of Chemistry and Biochemistry, Georgia Institute of Technology, Atlanta, GA, USA.
[5] Parker H. Petit Institute of Bioengineering and Bioscience, Georgia Institute of Technology, Atlanta, GA, USA.
[6] School of Biological Sciences, Georgia Institute of Technology, Atlanta, GA, USA.

***** Correspondence: alexandre.marques@usm.edu

1. Phosphorylation analysis: Threonine Kinase

The primary amino acid sequence of the Tc85-11 glycoprotein (accession: AAD13347.1) was analyzed for threonine phosphorylation potential using the NetPhos 3.1 server. The sequence is shown with predicted threonine phosphorylation sites (indicated by "T") in **Figure S1**.

```
>AAD13347.1 surface glycoprotein Tc85-11 [Trypanosoma cruzi]

    MSRRVFASAVLLLLLVLCCDRGATTAQVEKATDASTPSGSALTGAITAAG   #    50
    SASGSVELPQESILFVPQTTQVLQKTGTGSSGRDSFVSPSLVSAGGVIAA   #   100
    FAEGRINAKNTSPTESTKPSSDVVAEYIDSAWEWSTLVEKVKKKEWRART   #   150
    VLGKAEGNESFDVVVRHPTTIMKGNKVFLLVGSTALSVVNESWKEGSLEI   #   200
    KLVVGEVTKPTDSEPSKRIEWGEINSPLNGSTLAAHKGKLTECLASGGSG   #   250
    VLMEDGALVFSLMAVNEKNDGVYSMIIYSKDNGSTWALSEDMSPANCTDP   #   300
    RITEWEGSLLMIVDCENEQRVYESCDMGKTWTEAIGTLPGVWVNSQSEDY   #   350
    PEGVLRVDALITASIEDRKVMLYTQRGYASGGEAERALYLWVTDNNRSFF   #   400
    VGPVGMDNAVSGDLTSSLLYSDGKLHLLQRRGNSERSVISLSRLTEELST   #   450
    INSVLKTWAQNDAFFSNLSIPTAGLVAVLSNASASGDTWNDEYLCLNATV   #   500
    KNATKVKDGFQLQEPDSRAIWPVNTQGDNVRHISLSHNFTLVASVTIEEA   #   550
    PSEKTLLTAVLGNTEPPYIMRLSYTADNKWETMLKDEKTTRRSTWELKKE   #   600
    YQVALMLQGNKRSVYVDGELLGEEEVPLTGETPLEPFGFCFGACGEDDDG   #   650
    EEPSPEEIGKKPRVTVTNVFLYNRPLNSTEMTAIKDRKPVPKRAPEPQVK   #   700
    IVPNPVAPAVSAVPGPRELPAAPGRTTVGRTANTQHAPAGRLTSAGNEGT   #   750
    AREKGDGGANGDAGSAYGRELLPMLLLLGLWALATA                 #   800
%1  .......................TT.........................   #    50
%1  ...........................T......................   #   100
%1  ................T..................T.............T   #   150
%1  ..................TT..............................   #   200
%1  ........................................T.........   #   250
%1  ...............................................T..   #   300
%1  ..T..........................T......T.............   #   350
%1  .......................T..........................   #   400
%1  ..................................................   #   450
%1  ......T.........................................T.   #   500
%1  ...T....................T....................T....   #   550
%1  .............T.................T......TT...T......   #   600
%1  ............................T..T..................   #   650
%1  ..............T...................................   #   700
%1  ..........................T......T........T......T   #   750
%1  ....................................
```

Figure S1. Prediction of threonine phosphorylation sites in the Tc85-11 surface glycoprotein of Trypanosoma cruzi using NetPhos 3.1.*

Figure S2. Phosphorylation prediction. The figure displays the phosphorylation potential across the full sequence length. The green vertical bars represent predicted threonine phosphorylation sites, and the horizontal magenta line denotes the default threshold value for prediction confidence. One high-scoring site (T at position 594) within the motif RRSTW was predicted to be a potential phosphorylation target of Protein Kinase C (PKC), with a score of 0.684. This analysis suggests the presence of regulatory threonine phosphorylation sites that may contribute to the functional modulation of Tc85-11.

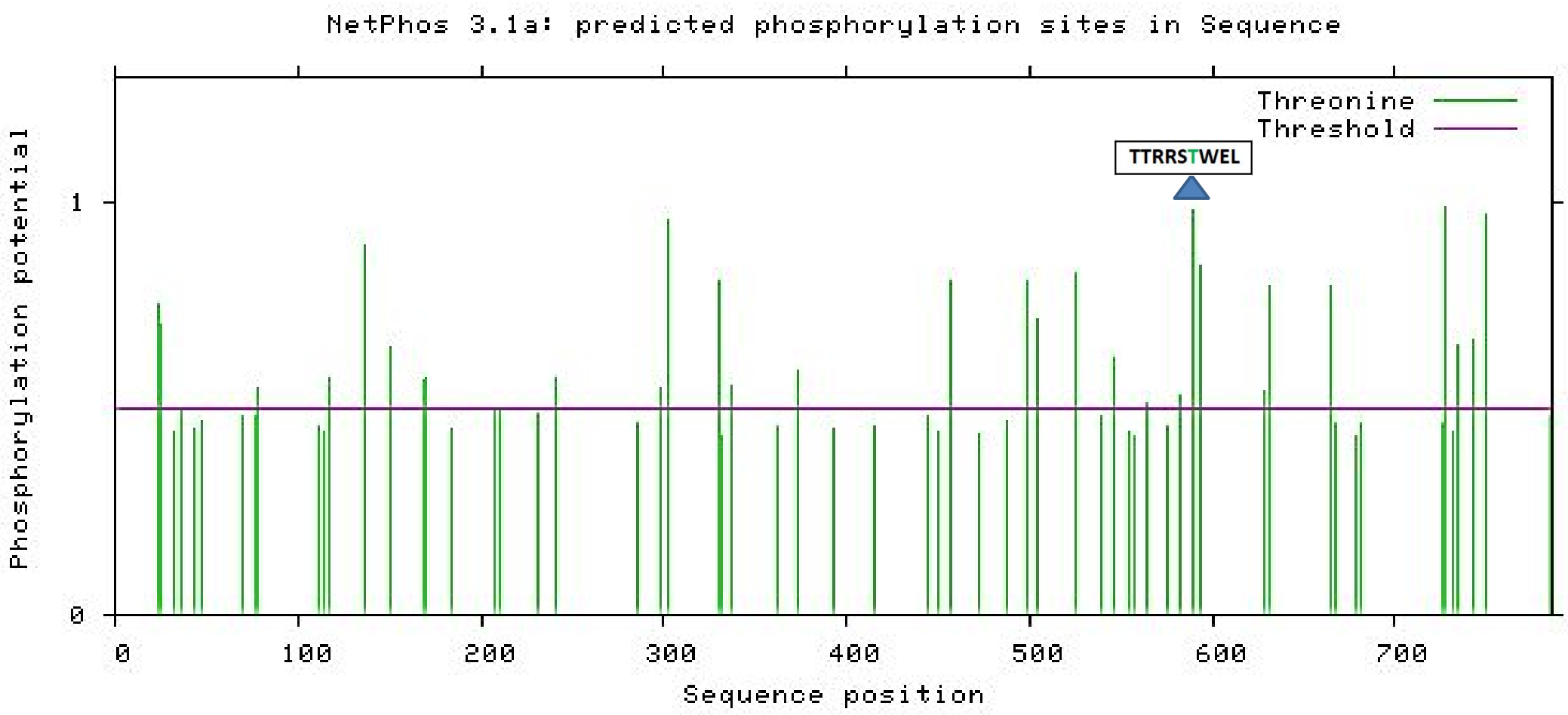

Position	Motif site	Sequence	Score	Kinase
594	RRSTW	TTRRSTWEL	0.684	PKC

Figure S2. Conservation and immunogenic potential of the RRSTW phosphorylation motif across *T. cruzi* trans-sialidase family members.

A conserved phosphorylation motif, a predicted protein kinase C (PKC) target, was identified at position 594 (motif = RRSTW, sequence = TTRRSTWEL)**.** Linear B-cell epitope prediction using ABCPred indicated a high antigenic score (0.62) at the core domain of the peptide, where the phosphorylated motif is located (**Table S1**). In parallel, structural modeling of the TSGII protein revealed that the RRSTW-containing region is surface-exposed, and conformational B cell epitope prediction using the ElliPro tool yielded a high antigenicity score (0.66), reinforcing its potential as an accessible immunogenic site (**Table S2**). Several peptides encompassing this motif are predicted to have strong binding affinities to human MHC class II alleles, HLA-DRB1*07:01, HLA-DRB1*08:02, HLA-DRB1*14:02, and HLA-DRB1*03:01, predominantly expressed in Latin American populations (**Table S3**). These findings support the potential of Tc85-derived peptides (Tc85pep) to elicit both humoral and cellular immune responses. Together, these results highlight the dual relevance of this conserved motif for phosphorylation-driven regulation and immune recognition in *T. cruzi* pathogenesis and vaccine design.

Table S1. Bioinformatic scores for B conformational cell exposition.

ABCpred Prediction = 0.51			Kolaskar	Emini: 1	ChouFas	Parker
Peptide	Length	Score	Antigenicity score	Accessibility score	β-turn score	Hydrophilicity score
KTTRRSTWELKKEYQVALML	20	0.62	0.95 < 0.98	1.0 < 1.81	0.89 < 0.93	1.70 < 2.58

Table S2. Evaluation of sequence conservation and surface exposure.

Conservation Treshold:70%		Surface Exposition	
Sequence matches at ≥30%	Maximun Identity	Ellipro	Score
70.49% (129/183)	100.00%	Exposed	0.66

Table S3. Affinity prediction to human MHC class II alleles.

allele	core peptide	peptide	IC_{50}	allele	core peptide	peptide	IC_{50}
DRB1*03:01	TRRSTWELK	EKTTRRSTWELKKEY	4920.92	DRB1*07:01			3629.77
DRB1*07:01	TTRRSTWEL	KDEKTTRRSTWELKK	2846.84	DRB1*08:02	TTRRSTWEL	DEKTTRRSTWELKKE	4815.37
DRB1*07:01	TRRSTWELK	EKTTRRSTWELKKEY	3012.16	DRB1*14:02			3603.74
DRB1*07:01	TTRRSTWEL	LKDEKTTRRSTWELK	3141.34	DRB1*03:01			4920.92
DRB1*07:01	RRSTWELKK	KTTRRSTWELKKEYQ	3220.77	DRB1*07:01	TRRSTWELK	EKTTRRSTWELKKEY	3012.16
DRB1*07:01	TTRRSTWEL	DEKTTRRSTWELKKE	3629.77	DRB1*08:02			
DRB1*08:02	RRSTWELKK	EKTTRRSTWELKKEY	4032.21	DRB1*14:02			2984.71
DRB1*08:02	RRSTWELKK	KTTRRSTWELKKEYQ	4058.38	DRB1*07:01	TTRRSTWEL	EKTTRRSTWELKKEY	2846.84
DRB1*08:02	TTRRSTWEL	KDEKTTRRSTWELKK	4216.98	DRB1*08:02			4216.98
DRB1*08:02	TTRRSTWEL	DEKTTRRSTWELKKE	4815.37	DRB1*14:02			3325.09
DRB1*08:02	TTRRSTWEL	LKDEKTTRRSTWELK	4907.26	DRB1*07:01			3220.77
DRB1*14:02	TTRRSTWEL	EKTTRRSTWELKKEY	2984.71	DRB1*08:02	RRSTWELKK	KTTRRSTWELKKEYQ	4058.38
DRB1*14:02	RRSTWELKK	KTTRRSTWELKKEYQ	3089.43	DRB1*14:02			3089.43
DRB1*14:02	TTRRSTWEL	KDEKTTRRSTWELKK	3325.09	DRB1*07:01			3141.34
DRB1*14:02	TTRRSTWEL	DEKTTRRSTWELKKE	3603.74	DRB1*08:02	TTRRSTWEL	LKDEKTTRRSTWELK	4907.26
DRB1*14:02	TTRRSTWEL	LKDEKTTRRSTWELK	3758.9	DRB1*14:02			3758.9

Figure S3. Serum dilution from immunized animals with TC85pep

Figure S3. IgG ELISA analysis against plated biotinylated Tc85pep for individual mice immunized with PP7-Tc85pep (20 µg) at week 3, establishing a titer of $3.2x10^3$.

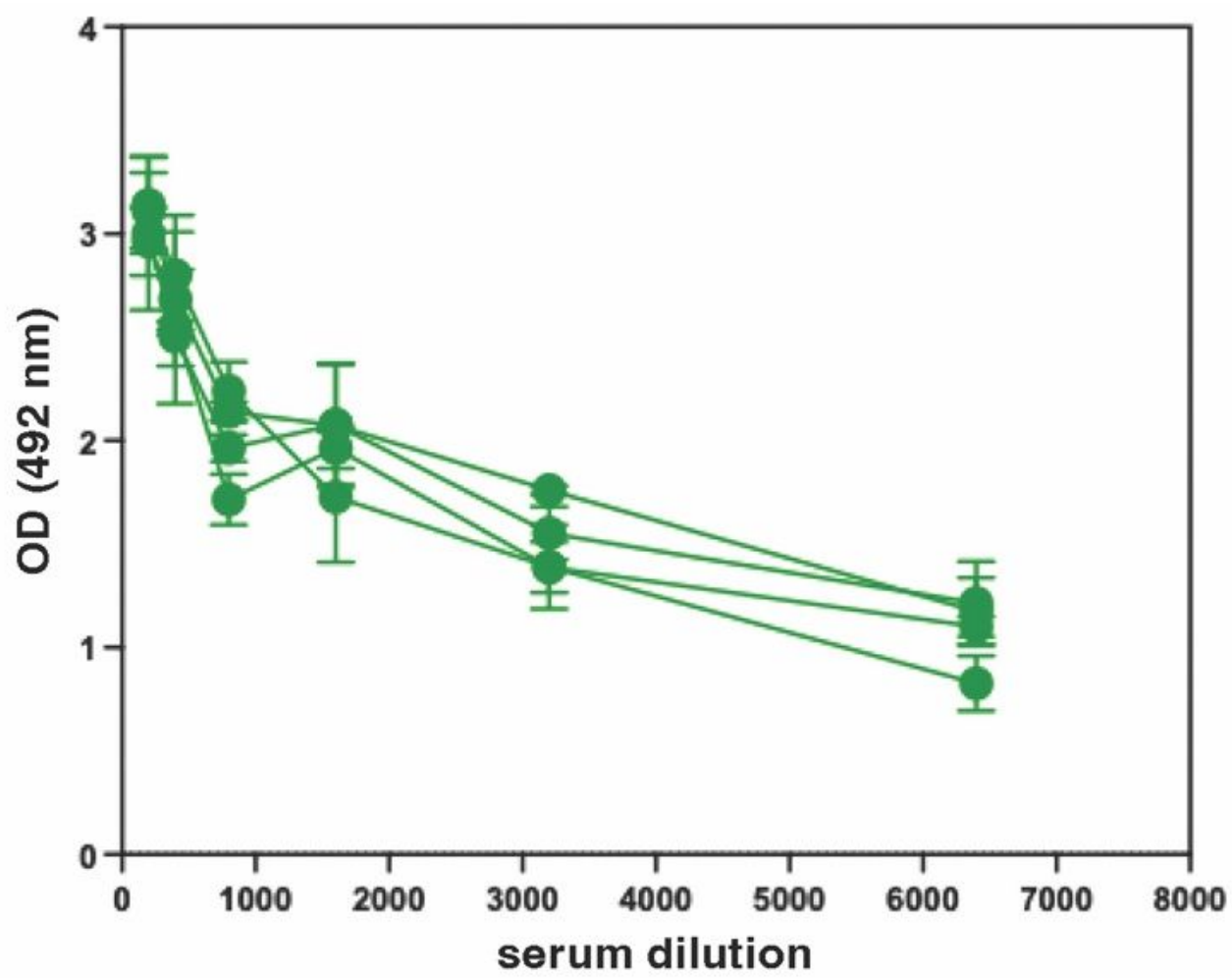

Supplement: Supplementary file 1 [file ao6c04457_si_001.pdf]
